# Proliferation assay amplification by IL-2 in model primary and recall antigen systems

**DOI:** 10.1186/1756-0500-7-662

**Published:** 2014-09-20

**Authors:** Amy SM Kennell, Keith G Gould, Myer R Salaman

**Affiliations:** Department of Immunology, Imperial College School of Medicine, London, UK

**Keywords:** T lymphocyte proliferation, IL-2, Primary response, Keyhole limpet haemocyanin, Pokeweed mitogen

## Abstract

**Background:**

It can be difficult to register a weak proliferative response of T lymphocytes to an antigen, particularly in a simple culture system of peripheral blood mononuclear cells (PBMC). Here we assess the usefulness of the cytokine IL-2 in amplifying such a response.

**Methods:**

PBMC from healthy donors were cultured in the presence or absence of keyhole limpet haemocyanin (KLH), an antigen to which people have not been previously exposed. IL-2 was added from the beginning or on the fifth day of culture. Proliferation was determined by incorporation of tritiated thymidine at eight days. The recall antigen, tuberculin PPD, provided a positive control.

**Results:**

IL-2 added at the beginning of culture can induce extremely high levels of proliferation even in the absence of antigen. However, when added on the fifth day it allowed the clear observation of a proliferative response to KLH that was barely detectable in its absence. Added late it was similarly able to boost low responses to PPD and to the mitogens lipopolysaccharide and poly(I:C), but it had no such effect with pokeweed mitogen.

**Conclusions:**

IL-2 added late in culture is highly effective in increasing the sensitivity of T lymphocyte proliferative assays.

## Background

It can sometimes be difficult to identify the presence of antigen-reactive T lymphocytes by assaying for antigen-induced proliferation of the cells. There are many reasons why this may be so. The number of reactive cells may be too small. Moreover, they may be in an anergic state or under the influence of regulatory T cells. There may be 'noise', that is the proliferation in culture of cells other than those being assayed. The problem may also be one of inappropriate antigen presentation or simply that the culture conditions are sub-optimal.

Some of these problems may be circumvented by purification of cell populations but this will inevitably detract from ease of assay. Another and attractive approach is to make use of the ability of the cytokine IL-2 to interact with T lymphocytes and augment cell division only after the lymphocytes have first been activated by recognition of antigen [1]. The activated cells express a high affinity receptor for IL-2 and proliferate in response to that cytokine. Although much use has been made of this property in the development of T cell lines and clones [2], it has not been widely used for assay amplification [3, 4]. Other cytokines have been considered for such amplification [4] (see Discussion), but IL-2 is by far the best understood and most readily available cytokine with the required property.

It is notoriously difficult to obtain a primary proliferative response to an exogenous antigen in a simple culture of peripheral blood mononuclear cells (PBMC) [5, 6]. In this paper we have attempted to get around the difficulties experienced with PBMC by amplifying proliferation through the addition of IL-2. We have studied the effect of added IL-2 on the response to keyhole limpet haemocyanin (KLH), an antigen with which people will have had no previous contact. By way of a positive control we have also tested the effect of IL-2 on the recall response to tuberculin PPD, a response which is not in need of amplification.

## Methods

### Antigens and mitogens

Keyhole limpet haemocyanin (KLH) was obtained from Sigma, Poole, UK (H7017). The 20 mg samples were taken up in 2 ml of sterile distilled water and diluted further with phosphate buffered saline (PBS) to 1 mg/ml. This was aliquoted and stored at -20°C.

Purified protein derivative (PPD), a freeze dried preparation of bovine tuberculin, was obtained from the Veterinary Laboratories Agency, Addlestone, UK. It was dissolved at 1 mg/ml in RPMI medium (see below) and sterilised by filtration. Aliquots were stored at -20°C.

Lipopolysaccharide (LPS), derived from E.coli 055:B5, came from Sigma (L-6529) as did pokeweed mitogen and poly(I:C).

### Culture of blood mononuclear cells (PBMC)

Blood was taken from healthy staff in the Department of Immunology at the St Mary's Campus of Imperial College School of Medicine. Fully informed verbal consent was obtained from all blood donors; the study design and consent was approved by St Mary's Local Research Ethics Committee. The Ethics Committee did not ask for the verbal consent to be recorded.

Samples (20 ml) were donated on multiple occasions by one male donor (M1) and five females (F1 - F5). The blood was taken into preservative-free heparin (20 units/ml), and PBMC were obtained by centrifugation on Histopaque-1077 (Sigma) of the blood diluted 1:1 with RPMI-1640 (Dutch Modification, Sigma R7638). The separated cells were taken up in culture medium, RPMI medium supplemented with 2 mM L-glutamine, 10 μg/ml gentamycin (Mayne Pharma, Warwickshire, UK) and 5% human AB serum (Sigma H-4522) at 0.15 × 10^6^ cells/ml.

Aliquots (1 ml) of the cells received 10 μl of appropriately diluted antigen/mitogen as required. Five replicate cultures of 0.18 ml were then set up from each aliquot in 96-well round-bottom culture plates (Nunc, Roskilde, Denmark). One control group without any antigen/mitogen was set up on each plate and up to 3 plates were set up on a given day. Plates were incubated in a humid atmosphere of 5% CO_2_ at 37°C for 8 days. After 5 days of culture all wells (unless indicated) received 5 μl of IL-2 to give a final concentration of 60 international units (i.u.)/ml. Recombinant human IL-2 was a kind gift from the Cetus Corporation (Emeryville, CA, USA).

Eighteen hours before termination of culture each well received 0.5 μCi of [*methyl*-^3^H] thymidine at 2 Ci/mmol (TRA310; Amersham Biosciences, Little Chalfont, UK). Uptake of thymidine into DNA was determined using a Packard system (Perkin Elmer, Mass. USA) involving filtration of lysed cells onto 96-well filter plates (UniFilter GF/C), addition of scintillant (MicroScint O) to each well followed by counting in a TopCount. The geometric mean of the five replicates was calculated. The value of the factor which on multiplication and division of the mean gives the range of one standard deviation generally fell between 1.1 and 2.2.

## Results

With the objective of increasing the sensitivity of the lymphocyte proliferation assay, we began by testing the effect of adding IL-2 to cultures in the absence of antigen. Table 1 shows five experiments in which IL-2 was added either at the start of the eight days of culture or after five days. In each experiment the mean uptake of tritiated thymidine at the end of culture was greatly enhanced when IL-2 had been present from the beginning. This was particularly marked for donors M1 and F1, the high level of response to IL-2 being confirmed in repeat experiments with these donors. Other female donors showed more modest enhancement.

**Table 1:** **Spontaneous lymphocyte proliferation in the presence of IL-2 added either at the beginning or towards the end of culture**

	Lymphocyte proliferation (cpm)				
Donor	No IL-2	IL-2 (60 i.u./ml) added		IL-2 (600 i.u./ml) added	
		Day 0	Day 5	Day 0	Day 5
M1	67	10306	-	28138	206
F1	66	17645	221	46194	238
F2	127	2091	160	-	-
F4	92	934	125	-	-
F5	105	866	128	-	-

PBMC from one male and four female donors were cultured at 0.15 × 10^6^ cells/ml for eight days. IL-2 at a final concentration of 60 or 600 i.u./ml was added either at the start of culture or after 5 days. Tritiated thymidine was present for the final 18 h of culture and mean values of thymidine uptake are shown.

When, however, addition of IL-2 was delayed until Day 5 only a small increase in baseline incorporation occurred. As will be seen, Day 5 addition of IL-2 was successful in boosting proliferation in the presence of antigen and therefore became the adopted procedure. The level of IL-2 chosen was 60 i.u./ml. Although 600 i.u./ml added from the beginning appeared more effective than 60 i.u./ml in the absence of antigen (Table 1), there was little difference in their effects with or without antigen when added on Day 5.

The amplified assay was tested using KLH as an antigen to which the donors had not been exposed previously (Table 2). KLH was tested at levels from 0.01 to 10 μg/ml but in any one experiment the concentration range did not exceed 100-fold. In the absence of IL-2, antigen responses were clearly too small and inconsistent to allow any definite conclusions. By contrast, in the presence of IL-2, clear-cut responses to KLH were seen at both 1 and 10 μg/ml, with evidence that the response peaks at about 1 μg. Even at 0.1 μg/ml responses could be detected.

**Table 2:** **IL-2 amplification of proliferation assay**

	Lymphocyte proliferation (cpm)											
	KLH (μg/ml)					KLH (μg/ml) + IL-2					PPD	PPD + IL-2
Donor	No antigen	0.01	0.1	1	10	No antigen	0.01	0.1	1	10		
M1	148	-	203	568	376	175	-	1981	2676	2004	6358^c^	17702
M1	72	70	90	104	-	163	437	1253	1070	-	1426^b^	8431
M1	108	-	113	128	279	221	-	429	1732	1178	419^a^	4846
F2	71	90	139	1241	-	123	212	380	2969	-	9233^c^	16818
F3	83	-	93	239	-	115	-	569	1163	-	216^b^	1691
F4	90	102	86	175	-	148	124	283	521	-	965^b^	5284

Mean values of thymidine uptake are shown for one male and three female donors in a total of six experiments. PBMC were cultured at 0.15 × 10^6^ cells/ml. IL-2 at 60 i.u./ml was added, where indicated, after 5 days. KLH or PPD were present from the start of culture as shown. PPD concentration in the different experiments is indicated by (a) 0.1, (b) 1 or (c) 10 μg/ml.

In each experiment the response to tuberculin PPD was used as a positive control. Provided that the donor is tuberculin-positive and a sufficiently high concentration of PPD is used there is no need for exogenous IL-2 in order to get a good response. Nevertheless, proliferation was strongly enhanced by IL-2 in each case (Table 2). If the effectiveness of IL-2 is expressed as the ratio of mean thymidine incorporation in the presence of PPD and IL-2 over mean incorporation with PPD alone, when the latter is high (first and fourth experiments) the enhancement ratio was around 2–3 fold. But when the response without IL-2 was more modest, the ratio ranged from 5–12. The corresponding values for KLH were expressed as geometric mean ratios at each level of the antigen. The mean values for donor M1 were used to calculate an overall mean for the four donors. Mean ratios were between 4–5 for KLH concentrations of 0.1, 1.0 or 10 μg/ml. Thus, where PPD responses in the absence of IL-2 were comparable to those of KLH, the enhancing effect of IL-2 appears at least as great for PPD as it was for KLH.

Several experiments of the type reported in Table 2 were carried out using lipopolysaccaride (LPS) in place of antigen. In the presence of IL-2 clear responses were observed at LPS levels down to at least 0.001 μg/ml, though the magnitude of the responses was not great. Even at 1 μg/ml the level of response was only around 1000 cpm. The IL-2 enhancement ratios were comparable to those seen with KLH, and this was also true of responses to poly(I:C) (0.2 - 2 μg/ml). Pokeweed mitogen was the only stimulatory agent used not to show clear IL-2 enhancement. The results for four different donors in the presence of pokeweed mitogen (10 μg/ml) were 8690 cpm (IL-2 absent)/8453 cpm (IL-2 present), 5832/9320, 23089/25713, and 2245/1403, giving a mean enhancement ratio of 1.02.

## Discussion

In this paper we have demonstrated the ability of added IL-2 to enhance low proliferative responses to KLH in a PBMC system. It is most important to add the IL-2 towards the end of the culture period. In confirmation of earlier studies by Taylor et al. [7], added from the beginning it induces high levels of proliferation in the absence of antigen because of the presence of activated T cells circulating in the blood. In the absence of IL-2 these cells evidently either die or lose their active status during the course of incubation. Preliminary data suggest that exceptionally high responses to IL-2 added from the beginning may be a characteristic of particular donors rather than being due to an acute infection. A similar assay system involving late addition of IL-2 has been proposed for use with mouse lymph node cells [8].

With IL-2 added after 5 days there was little effect on background proliferation and we were able to see a proliferative response to KLH at levels from 0.1 to 10 μg/ml (peaking at 1 μg) that was not observable in the absence of IL-2. Enhancement by IL-2 was also seen with the recall antigen PPD, the magnitude of the effect being similar to that for KLH when the response in the absence of IL-2 was low. It is of course well known that the proliferative response to PPD is a T cell response and this appears also to be the case for KLH [9]. Moreover, the T cell nature of these responses is further supported by the finding in preliminary experiments of inhibition of the KLH and PPD responses by antibodies against co-stimulatory molecules B7-1 and B7-2 [10]. It seems then that IL-2 enhancement can play a vital role where T cell proliferation is otherwise too low for assay purposes.

The mitogenic effects of LPS and poly(I:C) were likewise augmented by IL-2 to a similar extent as for KLH. This observation may suggest that the predominant proliferating cell here was the T cell, and these agents are known to be able to interact with T cells either directly or through their stimulation of other cell types [11, 12]. It would be expected that the magnitude of the IL-2 effect is independent of the mechanism of T cell activation. The failure of pokeweed mitogen to show IL-2 augmentation was surprising as this agent is directly mitogenic for both B and T cells [13] and appears to have a role in promoting B cell growth [14]. The explanation may lie in an exceptionally strong IL-2 response induced in PBMC by pokeweed mitogen [15], making exogenous IL-2 redundant.

The level of IL-2 used for amplification (60 i.u. or 3.3 ng/ml) appears high in relation to physiological levels. PBMC from BCG-vaccinated infants stimulated with PPD produced a mean of 17 pg/ml [16] and similar values with adult cells have been obtained for PHA stimulation [15]. A wide range of serum levels have been reported from undetectable to low nanogram quantities per ml. Clearly any IL-2 provided by the 5% serum in our cultures is not sufficient to induce significant proliferation.

Munier and colleagues [4] have investigated a similar approach for boosting low responses in PBMC to viral antigens. Their aim was to boost low secondary responses rather than to enable a primary response to be detected. Proliferation was assessed by a flow cytometric technique and, as might be expected, when IL-2 was added after three days of a ten day culture major enhancement occurred in the absence of viral antigen. Background proliferation was reduced when IL-2 was added after five days, but the best conditions for augmentation of responses were obtained not with IL-2 but with a combination of IL-15 and IL-21 added after five days.

We have seen that IL-2 added late in culture of PBMC is excellent at boosting antigen-induced T cell proliferation. That the procedure enables the measurement of a response to KLH and augments the response to PPD implies that it is effective in both primary and secondary responses. However, it will be important to confirm the action on primary responses using highly purified antigen systems. The procedure should be useful in studying the immune response to microorganisms including the field of vaccine development. Also, if the efficacy in primary response is confirmed, it could be applied to the study of the pathogenesis of autoimmune disease where the self-antigens involved may never under normal conditions interact with lymphocytes to induce either tolerance or an immune response [17]. Such purified self-antigens should in principle give rise to a primary response in cultures of PBMC from healthy donors as well as producing a secondary response in patients.

## Conclusions

This study demonstrates the striking ability of IL-2 added late in culture of PBMC to stimulate antigen-initiated T lymphocyte proliferation. Such an approach should be of great help where weak antigen responses are being assayed, in particular in the fields of infectious and autoimmune disease.
